# Utilizing multimodal mass spectrometry imaging for profiling immune cell composition and N-glycosylation across colorectal carcinoma disease progression

**DOI:** 10.3389/fphar.2023.1337319

**Published:** 2024-01-11

**Authors:** Lyndsay E. A. Young, Paul J. Nietert, Rachel Stubler, Caroline G. Kittrell, Grace Grimsley, David N. Lewin, Anand S. Mehta, Chadi Hajar, Katherine Wang, Elizabeth C. O’Quinn, Peggi M. Angel, Kristin Wallace, Richard R. Drake

**Affiliations:** ^1^ Department of Cell and Molecular Pharmacology and Experimental Therapeutics, College of Medicine, Medical University of South Carolina, Charleston, SC, United States; ^2^ Hollings Cancer Center, Medical University of South Carolina, Charleston, SC, United States; ^3^ Translational Science Laboratory, Hollings Cancer Center, Medical University of South Carolina, Charleston, SC, United States; ^4^ Department of Public Health Sciences, College of Medicine, Medical University of South Carolina, Charleston, SC, United States; ^5^ Department of Regenerative Medicine and Cell Biology, College of Medicine, Medical University of South Carolina, Charleston, SC, United States; ^6^ Department of Pathology and Laboratory Medicine, College of Medicine, Medical University of South Carolina, Charleston, SC, United States

**Keywords:** colorectal carcinoma, adenoma, N-glycosylation, imaging mass spectrometry, spatial biology

## Abstract

Colorectal cancer (CRC) stands as a leading cause of death worldwide, often arising from specific genetic mutations, progressing from pre-cancerous adenomas to adenocarcinomas. Early detection through regular screening can result in a 90% 5-year survival rate for patients. However, unfortunately, only a fraction of CRC cases are identified at pre-invasive stages, allowing progression to occur silently over 10–15 years. The intricate interplay between the immune system and tumor cells within the tumor microenvironment plays a pivotal role in the progression of CRC. Immune cell clusters can either inhibit or facilitate tumor initiation, growth, and metastasis. To gain a better understanding of this relationship, we conducted N-glycomic profiling using matrix-assisted laser desorption-ionization mass spectrometry imaging (MALDI-MSI). We detected nearly 100 N-glycan species across all samples, revealing a shift in N-glycome profiles from normal to cancerous tissues, marked by a decrease in high mannose N-glycans. Further analysis of precancerous to invasive carcinomas showed an increase in pauci-mannose biantennary, and tetraantennary N-glycans with disease progression. Moreover, a distinct stratification in the N-glycome profile was observed between non-mucinous and mucinous CRC tissues, driven by pauci-mannose, high mannose, and bisecting N-glycans. Notably, we identified immune clusters of CD20^+^ B cells and CD3/CD44+ T cells distinctive and predictive with signature profiles of bisecting and branched N-glycans. These spatial N-glycan profiles offer potential biomarkers and therapeutic targets throughout the progression of CRC.

## Introduction

Colorectal cancer (CRC) is the third leading cause of cancer in men and women in the US and the second leading cause of cancer death (Siegel et al., 2019; Siegel et al., 2020). Over the past 2 decades, CRC incidence and mortality rates have declined in older adults, partly due to increased screening (Siegel et al., 2020). However, during the same period, there has been an increase in CRC incidence among younger individuals (under 50 years of age), underscoring the need to identify novel biomarkers (Sifaki-Pistolla et al., 2022). One specific area of interest is mucinous colorectal cancer, a distinct form of CRC found in 10%–15% of patients, with most diagnosed at advanced stages (Siegel et al., 2019). The prognosis of mucinous colorectal adenocarcinoma to non-mucinous colorectal adenocarcinoma is debatable but understanding the molecular differences between the cancer subtypes warrants investigation.

Mounting evidence suggests tumor progression and recurrence are strongly influenced by the tumor microenvironment (TME). The TME landscape encompasses a multitude of cell types with specific roles in tumor progression and eventual metastasis. In recent years, the impact of the TME has gained attention in CRC, prompting the extensive analysis of clinical trials to assess immune-cell infiltration as prognostic and predictive markers. These promising results have been achieved with the use of immune checkpoint inhibitors in CRC in subgroups of patients. These findings suggest a larger role for understanding the TME for therapeutic interventions. Intrinsic to the TME are glycosylated proteins, lipids and sugar polymers at the cell surface and surrounding stroma. Glycosylation is a common post-translational modification with at least half of all human proteins modified by adding a carbohydrate group. (Apweiler et al., 1999). Glycoproteins play critical functional roles in molecular transport, enzymic reactions, cell adhesion, immune effector function, protein folding, and more (Reily et al., 2019; Varki et al., 2022). The normal colonic mucin layer consists of highly glycosylated proteins that provide essential functions including lubrication of food, cell signaling, and protection of host from pathogens and toxins. Aberrant glycosylation is increasingly recognized as a hallmark of carcinogenesis active at all phases, including initiation, progression, and metastatic expansion (Thomas et al., 2021). This highlights the potential of N-glycans as biomarkers across the neoplastic continuum (Munkley and Elliott, 2016). Studies on N-glycosylation in colorectal carcinogenesis have primarily focused on comparing serum and or cancer tissues samples to healthy controls or surrounding normal tissue (Balog et al., 2012; Holst et al., 2013; Sethi et al., 2015; Holm et al., 2020; Boyaval et al., 2021; Coura et al., 2021; Boyaval et al., 2022). Several unique N-glycans signatures such as high-mannose, sulfated glycans, and paucimannose groups are associated with colon cancer progression and metastasis (Balog et al., 2012; Holst et al., 2015; Boyaval et al., 2021). However, few studies have examined N-glycosylation in early carcinogenesis comparing preinvasive to invasive lesions (Kaprio et al., 2015; Boyaval et al., 2022). No studies have yet compared N-glycan expression encompassing normal healthy patients, preinvasive lesions, and cancerous colon tissues at different stages.

The most classical and commonly-used therapy in CRC is chemotherapy. Treatments with chemotherapeutics such as 5-fluorouracil (5-FU), oxaliplatin (L-OHP), vincristine (VCR), doxorubicin (DOX), cisplatin (CDDP)and irinotecan (CPT-11) have all contributed to prolonged survival in patients with advanced CRC (Cunningham et al., 2010). Despite these improvements, more than 90% of patients with metastatic disease have a poor prognosis due to drug resistance (Longley and Johnston, 2005). Various strategies including drug repurposing, gene therapy and protein inhibitors have been employed or investigated to combat drug resistance (Ma et al., 2023). A subset of CRC patients with metastastic disease receive additional targeted and immunotherapies in efforts of better outcomes (Elez and Baraibar, 2022). Monoclonal antibodies such as Cetuximab, Bevacizumab and Pantiumumab directly target tumor antigens and have shown some efficacy in treatment of CRC (Lu et al., 2020). Additionally, three immune checkpoint inhibitors, pembrolizumab, nivolumab, and ipilimumab are FDA approved therapies for CRC (Weng et al., 2022).Additionally, immune cells in the TME have been shown to modulate cancer progression and represent attractive therapeutic targets. Glycoproteins represent the major share of marketed and clinical development phase therapeutic proteins. Glycans enhance thermal stability, provide protection from proteolysis, improve solubility, and inhibit aggregation of proteins (Li and Anjou, 2009). Dissection of the tumor glyco-code has identified glycosylation changes of the tumor, tumor microenvironment, and metastasis. Further, Instances of silylation status of tumor antigens demonstrate a direct impact on tumor infiltrating cells, driving an immune-inhibitory circuit (RodrÍguez et al., 2018). Cancer-associated glycans present on cancer cells have been shown to structurally impair the accessibility of protein targets to therapeutic antibodies (Peiris et al., 2017). For instance, the affinity of the therapeutic antibody AR20.5, which targets MUC1, has been demonstrated to be glycan-dependent (Movahedin et al., 2017). The critical impact of N-glycosylation on therapeutic efficiency underscores the importance of understanding the N-glycome of patient samples. N-glycoproteomic studies have identified several distinct peptides between tumor and adjacent tissues. Using a N-acetylglucosamine lectin, 17 proteins in tumor tissues compared to adjacent normal tissue (Li et al., 2013). Additional N-glycoproteomic work by Sethi and others identified a unique link between CRC stage, EGFR status and N-glycan features specific to EGFR (Sethi et al., 2015). Further, additional work has demonstrated MGAT5-mediated branched N-glycans are an immune checkpoint in colorectal cancer and are overexpressed during colorectal cancer carcinogenesis (Silva et al., 2020). Finally, glycosylation status shapes T-cell tumor immune response specifically with impacts on Treg activation, increases in T-cell receptor activation thresholds, and immune escape of tumor cells (Fernandes et al., 2023). These differences in N-glycoproteome may pave the way for more targeted therapeutic strategies.

For this study, a cohort of archived pathology tissues were assembled that represented normal colon epithelia, different classes of adenomas and polyps, and invasive colorectal carcinomas, including mucinous carcinoma. The tissues were assessed using N-glycan imaging mass spectrometry to define the compositions and differences of the N-glycans associated with disease progression (Powers et al., 2014; Drake et al., 2017; McDowell et al., 2023). Each tissue was annotated by pathologists to define glycans present in different cellular sub-types, including immune cell clusters. Finally, we then identified the cellular composition of these immune clusters using MALDI-IHC.

## Methods

### Materials

HPLC-grade water, xylene, citraconic anhydride, acetonitrile, and methanol were obtained from Fisher Scientific (Hampton, NH, SA). Ethanol was obtained from Decon Labs (King of Prussia, PA). Trifluoroacetic acid (TFA) and α-cyano-4-hydroxycinnamic acid (CHCA) were purchased from Sigma-Aldrich (St. Louis, MO, SA). Louis, MO). Peptide-N-glycosidase F (PNGase F) PRIME™ was obtained from N-Zyme Scientific (Doylestown, PA). H&E staining was performed using Cancer Diagnostics (Durham, NC, United States).

### Colorectal FFPE tissue samples and pathologic annotation

FFPE samples were provided by the Medical University of South Carolina Hollings Cancer Center Biorepository and Tissue Analysis Shared Resource with an Institutional Review Board-approved protocol to use clinically exempt, de-identified samples at 5-micron thin sections for H&E and N-glycan analysis. The samples included normal colonic tissues (normal, *n* = 6), adenomas and serrated lesions (polyps, *n* = 9), tubulovillous adenomas near adjacent carcinomas (tumor associated polyps, *n* = 8), and adenocarcinomas (cancers, *n* = 14). Patients contributing normal mucosa tissue samples had no evidence of colorectal polyps or cancer at the colonoscopy. For each sample, we abstracted information on age at diagnosis, sex, race, BMI at diagnosis, smoking status, and clinicopathologic features such as location, grade, stage, and histologic type.

The diagnostic GI pathologist (DL) independently reviewed all lesions after sectioning. A second pathologist used QuPath to annotate regions of interest (ROIs) for the whole slide image, including cancer-adjacent polyp ROIs within the CRCs, when present (Bankhead et al., 2017).

### Tissue preparation for MALDI-MSI

N-glycan profiling by MALDI-MSI was performed using a standardized protocol, as previously described (Powers et al., 2014; Drake et al., 2018). FFPE tissues were dewaxed by heating at 60°C for 1 h, washed in xylene, and rehydrated using a graded series of ethanol and water. Slides were antigen retrieved in citraconic anhydride buffer (25 µL citraconic anhydride, 2 µL 12 M HCl, 50 mL of HPLC water, pH 3.0 ± 0.5) for 20 min in a decloaking chamber at 95°C. Fifteen passes of 0.1 μg/μL PNGase F PRIME were applied using an M5 TM Sprayer (25 μL/min flow rate, 1,200 mm/min, 45°C, 3 mm offset, 10 psi nitrogen gas, HTX Technologies LC). Slides were incubated for 2 h at 37°C in preheated humidified chambers. After desiccation, 7 mg/mL CHCA matrix in 50% acetonitrile/0.1% TFA was applied using a TM Sprayer (10 passes, 100 μL/min flow rate, 1,300 mm/min, 79°C, 2.5 mm offset, 10 psi nitrogen). The slides were stored in a desiccator until analysis.

### MALDI-QTOF analysis of N-Glycans

Tissue samples were analyzed using a dual-source timsTOFfleX MALDI-QTOF mass spectrometer (Bruker) operating in positive mode. A 10 kHz SmartBeam 3D laser with a 20 µm laser spot size was run at a 40 µm raster with 300 laser shots per pixel to produce high-resolution images of N-glycan spatial localization in the m/z range of 700–4,000.

### Tissue preparation for MALDI-IHC

Multiplexed antibody profiling of tissues and immune cell composition was done using Miralys™ MALDI HiPLEX-IHC (Ambergen), as previously described (Yagnik et al., 2021). Briefly, FFPE tissues were dewaxed for 2 h, and rehydrated using xylene and ethanol washes. Slides were antigen retrieved in 1X Alkaline Retrieval Buffer for 30 min in a veggie steamer. Following a cooling of 30 min, the slides were placed in tissue block buffer (2% v/v Mouse serum, 2% v/v Rabbit serum and 5% w/v BSA in 0.05% TBS OBG) for 1 h. Humidity chambers were prepared by preheating at 37°C for 30 min and immediately followed by 30 min in a 4°C room. Working probe mix was prepared with the 3–3.75 ug/mL of each antibody into tissue blocking buffer and then filtered. 250 ul of each antibody was applied to the tissue and a coverwell incubation chamber (Invitrogen) was applied to cover the tissue. Slides were incubated at 4°C in the humidity chambers on a slow rotating platform covered in foil overnight. The following day, each slide was processed in 1X TBS and 50 mM ammonium bicarbonate for several washes and then dried in a covered desiccator for 1.5 h. Slides were placed in an AmberGen Light Box for 10 min to allow for cleavage of the tag from the antibody. Finally, slides were coated in 10 mg/mL CHCA matrix in 70% acetonitril/0.1% TFA using a TM Sprayer (8passes, 100 μL/min flow rate, 1350 mm/min, 60°C, 3 mm offset, 10 psi nitrogen). Recrystallization by 5% IPA followed for 1°min in 60°C oven. The slides were desiccated until analysis. Tissue samples were analyzed using a dual-source timsTOFfleX MALDI-QTOF mass spectrometer (Bruker) operating in positive mode. A 10 kHz SmartBeam 3D laser with a 20 µm laser spot size was run at a 40 µm raster with 300 laser shots per pixel to produce high-resolution images of tag spatial localization in the m/z range of 800–1,800.

### MS data processing and analysis

MS data were imported into SCiLS Lab software 2022b Pro (Bruker) for analysis in the mass range of m/z 700–4,000, or 800–1,850 and the spectra were normalized to the total ion count (ICR Noise Reduction Threshold = 0 95). N-glycan peaks were manually selected based on the theoretical mass values within ±5 ppm of an in-house database of known N-glycans and prior MS/MS collision induced fragmentation characterizations (McDowell et al., 2021). Photocleaveable tag peaks were selected from known mass tag m/z values provided by AmberGen Inc.

### Statistical considerations

Demographics were compared among the sample types (normal, polyp, cancer) using Kruskal-Wallis and Fisher’s exact tests, as appropriate. The relative abundances of the various glycan groupings (i.e., pauci-mannose, high-mannose, hybrid, biantennary, triantennary, tetrantennary), expressed as the percentage of total glycan abundance, were compared among different phases of carcinogenesis (normal, polyp, cancer-adjacent polyp, cancer) using generalized linear mixed models, which incorporated age as a covariate and random subject effects to account for within-patient clustering. Kruskal-Wallis tests were used to compare relative frequencies of the various glycan groupings among samples from patients at different cancer stages (I, II, III/IV). *p*-values <0.05 were considered statistically significant, and due to the hypothesis-generating nature of this study, no adjustment was made for multiple comparisons. All analyses were conducted using SAS V9.4 (SAS Institute, Cary, NC). Metaboanalyst 5.0 was used to generate the principal component analyses, dendogram, heatmaps, and mass correlation plots via PatternHunter.

## Results

**FIGURE 1:**
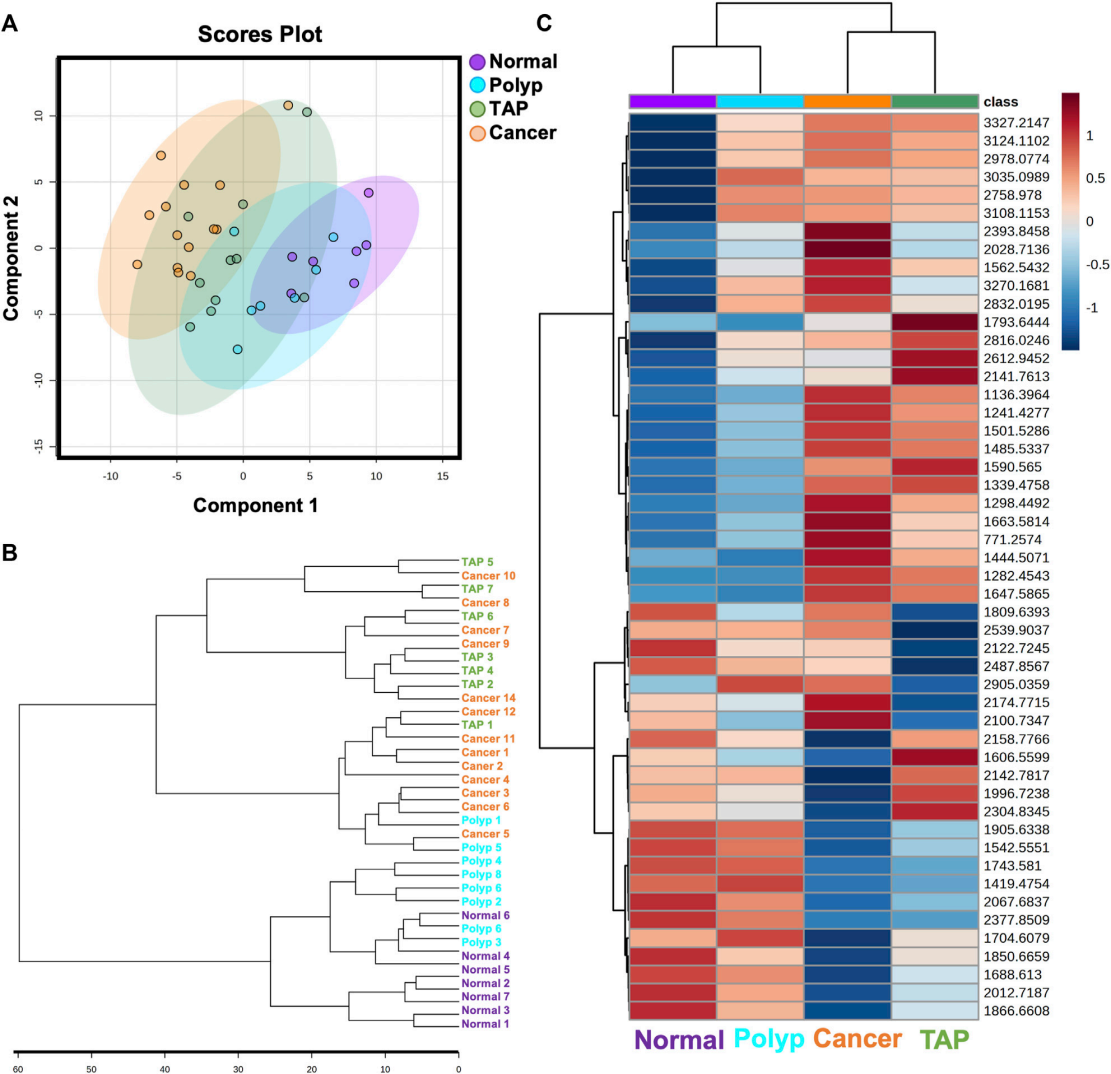
Overview of N-glycan profiles across normal and colorectal carcinoma patient samples. **(A)** Multivariate analysis of normal, polyp, tumor associated polyp, and cancerous patient samples displaying 95% confidence intervals. Each point represents one patient sample displaying all N-glycan features. **(B)** Unsupervised clustering analysis of N-glycans demonstrating stratification of samples by state of carcinogenesis. **(C)** Unbiased clustering heatmap analysis of the top 50 N-glycan features stratifying the groups.

Our study examined 30 colorectal specimens, including 7 normal colon specimens, 9 preinvasive lesions, and 14 cancers (Table 1). No significant differences in patient characteristics of ag, BMI, sex race or smoker status were identified by sample type at diagnosis (Table 1). To investigate the unique N-glycan profile, we utilized a previously established MALDI-MSI workflow for FFPE tissues (Powers et al., 2014). The peak intensities for nearly 100 N-glycan compositions (see Supplementary Table S1) were compiled for each tissue sample. Multivariate analysis comparing normal, polyp, tumor adjacent polyp tissue, and cancerous tissues demonstrated distinct N-glycome profiles for each sample group (Figure 1A; Supplementary Figure S1). Further hierarchical clustering identified two specific groupings of tumor adjacent polyps and cancerous tissues, and another for polyps and normal tissue, indicating a distinct N-glycome profiles of the top 50 significantly changed N-glycans (Figures 1B, C).

**TABLE 1:** Summary table of demographics for colorectal carcinoma and normal patient samples. Features include age, BMI, sex, race and smoker status. IQR: interquartile range.

	*Normal (n = 7)*	*Polyp (n = 9)*	*Cancer (n = 14)*	*p-value*
*Age (median [IQR])*	46 [39–56]	61 [52–71]	60 [50–68]	0.11
*BMI (median [IQR])*	25.7 [23.0–29.5]	29.6 [28.5–32.0]	23.2 [18.5–25.3]	0.38
*Sex*				0.80
*Male: n (%)*	2 (29)	4 (44)	6 (43)	
*Female: n (%)*	5 (71)	5 (56)	8 (57)	
*Race*				0.27
*White: n (%)*	5 (71)	6 (67)	7 (50)	
*Black: n (%)*	1 (14)	3 (33)	7 (50)	
*Hispanic: n (%)*	1 (14)	0 (0)	0 (0)	
*Smoker*				0.55
*Never: n (%)*	3 (43)	2 (40)	10 (71)	
*Former: n (%)*	2 (29)	2 (40)	3 (21)	
*Current: n (%)*	2 (29)	1 (20)	1 (7)	

**FIGURE 2:**
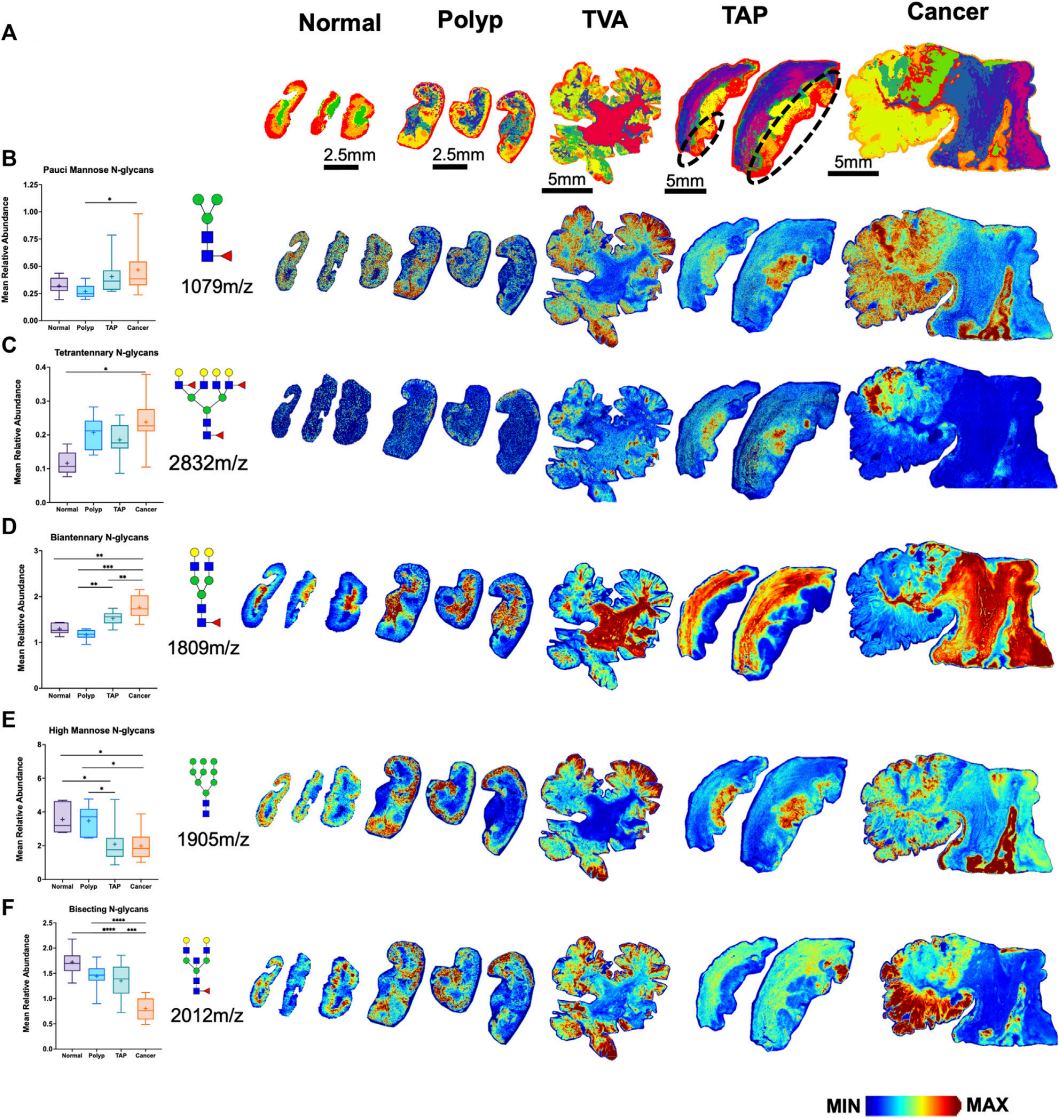
Distinct N-glycome signatures through CRC cancer progression. **(A)** Segmentation analysis by k-bisecting mean and Manhattan metrics of the N-glycome demonstrating spatial differences of N-glycans across the tissue. Relative Abundance of N-glycans across normal, polyp, TVA, TAP (annotated), and cancerous tissue with corresponding representative MALDI-MSI images. TVA was included to demonstrate malignant like features in this polyp. **(B)** Relative abundance of paucimannose glycans with representative images for paucimannose 1,079 m/z (Hex3HexNAc2Fuc1), **(C)** for tetraantennary 2,832 m/z (Hex7HexNAc6Fuc1Sia1), **(D)** for biantennary 1,809 m/z (Hex5HexNAc4Fuc1), **(E)** for high mannose 1,905 m/z (Hex9HexNAc2), **(F)** for bisecting 2012m/z (Hex5HexNAc5Fuc1). Intensity gradient from blue (least abundant) to red (most abundant). Scale bars are below images.*p < 0.05, **p < 0.01, ***p < 0.001, ****p < 0.0001 using mixed models that accounted for within-person clustering and adjusted for patient age.

Among the six glycan structural groups examined (i.e., paucimannose, high-mannose, biantennary, bisecting, and tetraantennary), six showed significant differences in age-adjusted relative expression by sample type with distinct histopathological locations as demonstrated by segmentation analysis (Table 2; Figure 2A). Levels of pauci-mannose N-glycans exhibited significantly higher in cancers compared to polyps while tetraantennary N-glycans were significantly higher in cancers compared to normal tissue (Figures 2B, C). The biantennary and hybrid glycan group also exhibited higher relative expression but only between normal samples and cancers, tumor adjacent polyps, and polyps compared to normal tissues (Figure 2D; Table 2). On the contrary, high-mannose and bisecting N-glycan groups were found to have relatively higher expression in polyps (and normal tissue) compared to tumor adjacent polyps and cancers (Figures 2E, F). No differences in glycan expression by sample type were observed for triantennary N-glycans (Table 2).

**TABLE 2:** Relative abundances* of various N-glycan classes at different states of carcinogenesis.

	Normal *n* = 7	Polyp *n* = 9	Tumor associated polyps *n* = 8	Cancer *n* = 14	Significant (*p* < 0.05) pairwise comparisons
Glycan grouping	Mean % (SD)	Mean % (SD)	Mean % (SD)	Mean % (SD)	
Pauci-mannose	0.35 (0.07)	0.30 (0.06)	0.42 (0.14)	0.47 (0.18)	^2^
High mannose	3.56 (0.84)	3.28 (1.03)	2.13 (1.20)	1.99 (0.83)	^1,2,5,6^
Bisecting	1.30 (0.12)	1.16 (0.13)	0.94 (0.21)	0.77 (0.2)	^1,2,5,6^
Hybrid	0.76 (0.09)	0.80 (0.16)	1.07 (0.17)	1.00 (0.17)	^1,2,5,6^
Biantennary	1.14 (0.11)	1.06 (0.15)	1.29 (0.09)	1.46 (0.17)	^1,2,3,6^
Triantennary	0.38 (0.05)	0.42 (0.04)	0.46 (0.11)	0.44 (0.1)	
Tetrantennary	0.20 (0.05)	0.27 (0.06)	0.28 (0.08)	0.24 (0.07)	^5^

*All measures represent relative abundance; thus the units for each are percentages. Significant (*p* < 0.05) pairwise comparisons obtained from general linear mixed models: ^1^ cancer vs. normal; ^2^ cancer vs. polyp; ^3^ cancer vs. tumor adjacent polyp; ^4^ normal vs. polyp; ^5^ normal vs. tumor adjacent polyp; ^6^ polyp vs. tumor adjacent polyp.

**FIGURE 3:**
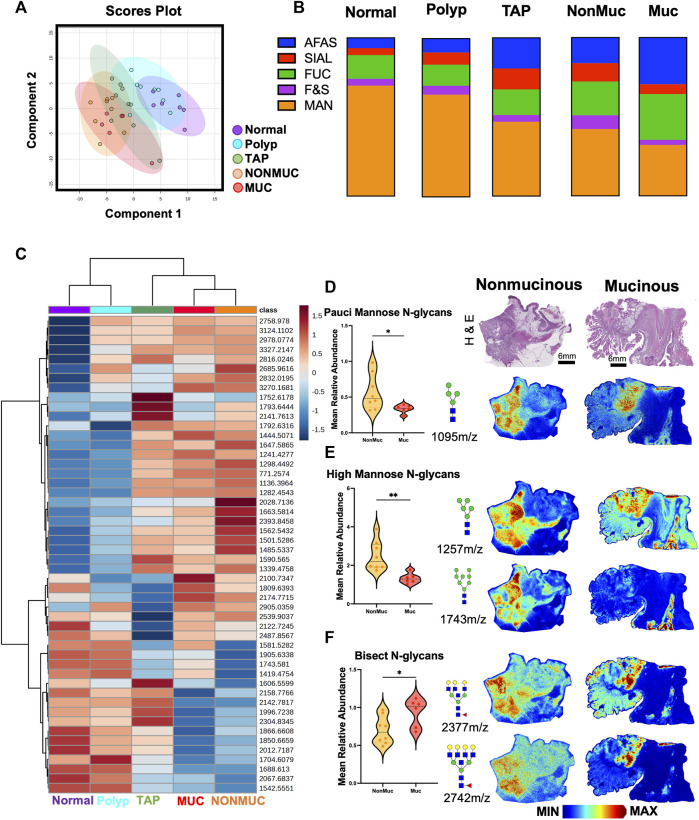
Distinct N-glycan features within non-mucinous and mucinous colorectal carcinoma samples. **(A)** Unsupervised clustering analysis of N-glycans demonstrating stratification of samples by state of carcinogenesis with specific distinction in mucinous vs. nonmucinous carcinoma tissues. **(B)** Overview of N-glycan class composition as percentages within each sample group. AFAS- A-fucosylated & A-sialylted, F&S- Fucosylated & Sialylated, FUC-Fucose, Man-Mannose, and SIAL-Sialylated. **(C)** Unbiased clustering heatmap analysis of the top 50 N-glycan features stratifying the groups. **(D)** Relative Abundance of pauci-mannose N-glycans across nonmucinous and mucinous cancerous tissue with corresponding representative MALDI-MSI image of 1095 m/z Hex4HexNAc2. **(E)** Relative Abundance of high mannose N-glycans across nonmucinous and mucinous cancerous tissue with corresponding representative MALDI-MSI image of 1,257 m/z Hex4HexNAc2 and 1,743 m/z Hex8HexNAc2. **(F)** Relative Abundance of bisecting N-glycans across nonmucinous and mucinous cancerous tissue with corresponding representative MALDI-MSI images of 2,377 m/z Hex6dHex1HexNAc6 and 2,742 m/z Hex7dHex1HexNAc7. Intensity gradient from blue (least abundant) to red most abundant. Scale bars are below images. *p < 0.05, **p < 0.01 using an Unpaired t-test. *n* = 6 mucinous, *n* = 9 nonmucinous samples.

Building upon our initial observation of distinct disease progression, we hypothesized distinct differences in N-glycan profile for mucinous and non-mucinous tumors. Multivariate analysis comparing normal, polyp, tumor adjacent polyps, mucinous and nonmucinous tissues demonstrated distinct N-glycome profiles for each sample group (Figure 3A). Remarkably, there are distinct differences in classification profiles across afucosylated and asialylated (AFAS), fucosylated and sialylated (F&S), fucosylated (Fuc), high mannose (Man) and sialylated (Sial) N-glycans (Figure 3B). Unsupervised hierarchial clustering analysis further demonstrated N-glycan changes through disease progression from normal to cancerous tissue as well as specific N-glycan differences between mucinous and non-mucinous samples (Figure 3C). This division between mucinous and nonmucinous tissues was notably driven by a decrease in pauci-mannose and high mannose N-glycans, with an increase in bisecting N-glycans (Figures 3D–F) Spatial distinctions between mucinous and nonmucinous cancerous tissue can be seen by accompanying representative images throughout normal tissue, the tumor, and the tumor microenvironment (Figures 2D–F). These differences prompted a more in-depth exploration of the histopathology of the cohort.

We also hypothesized stage-dependent differences in N-glycan signatures across cancerous tissues. Decreases in N-glycan percentages were identified for pauci-mannose and high-mannose N-glycans groups by cancer stage (I, II, III/IV). While no significant differences were identified in the percentage of N-glycan classes, various trends were apparent. Pauci-mannose expression trending decreases in early to later stage CRCs (Supplementary Figure S2). Specifically, decreases were noted in the annotated adenocarcinoma (iACa) regions (Supplementary Figure S2B) of each CRC tissue for 933 m/z Hex3HexNAc2, and 1,079 m/z Hex3dHex1HexNAc2 (Supplementary Figure S2C, D). In contrast, biantennary N-glycan profiles showed no significant trends in iACa regions across stage as seen in 1,955 m/z Hex5dHex2HexNAc4, 2,101 m/z Hex5dHex3HexNAc4, 2,158 m/z Hex5dHex2HexNAc5, and 2,449 m/z Hex5dHex2HexNAc5NeuAc1 (Supplementary Figure S3A, C–F). Additionally, trending increases were noted for the tetraantennary N-glycans of each CRC tissue for 1,891 m/z Hex3dHex1HexNAc6, 2,978 m/z Hex7dHex4HexNAc6, 3,051 m/z Hex8dHex2HexNAc7, and 3,343 m/z Hex8dHex4HexNAc7 (Supplementary Figure S4). Finally, trending decreases were noted for the high mannose N-glycans of each CRC tissue for 1,419 m/z Hex6HexNAc2, 1,581 m/z Hex7HexNAc2, 1,743 m/z Hex8HexNAc2, and 1,905 m/z Hex9HexNAc2 (Supplementary Figure S4). No other differences by stage or global differences in glycan groups by histologic type were found.

**FIGURE 4:**
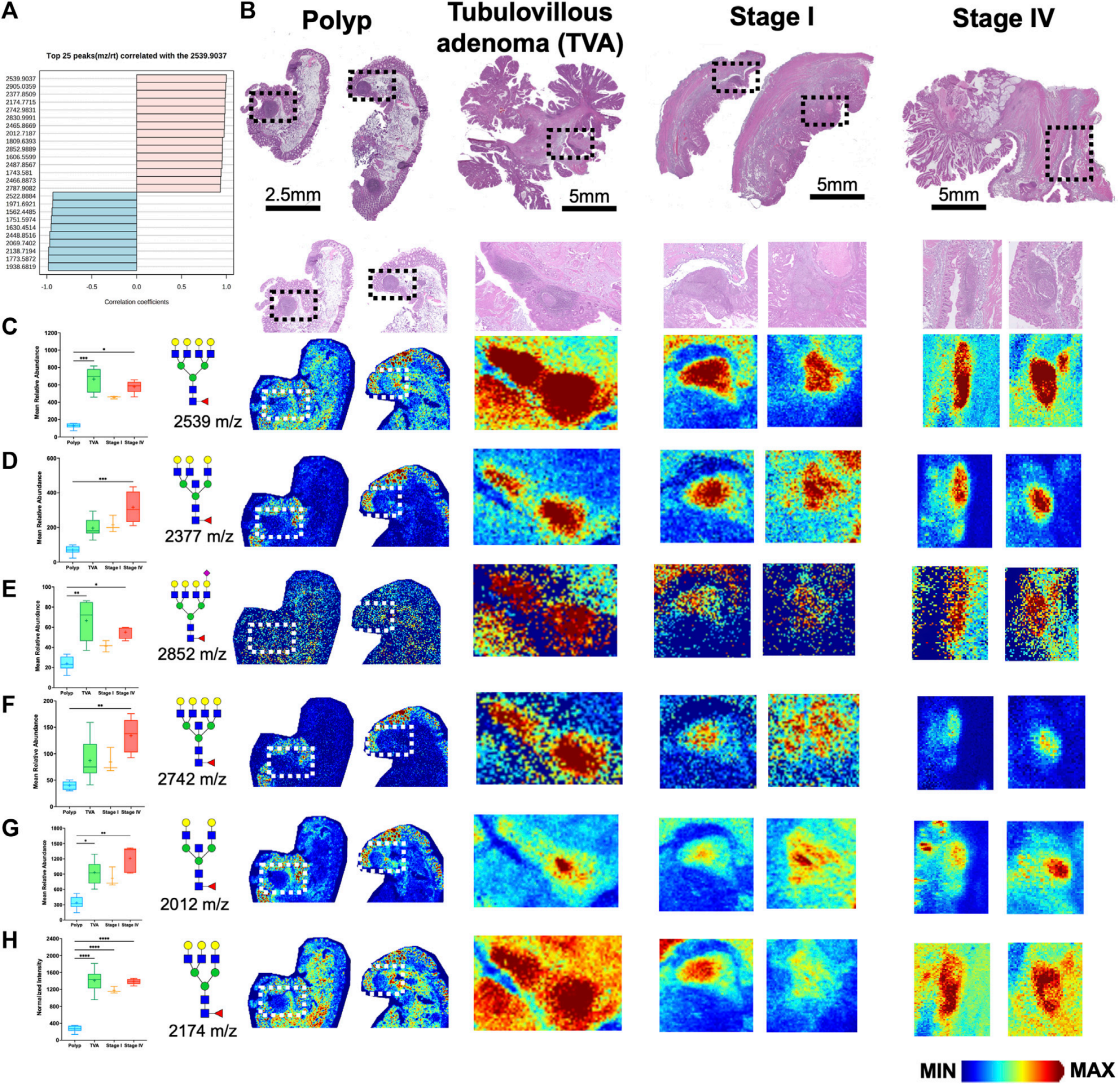
Immune clusters within the tumor microenvironment of CRC tissues exhibit enrichment of six N-glycans. **(A)** Top 25 N-glycans that correlated with 2,539 m/z Hex7dHex1HexNAc6. **(B)** H&E images of polyp, tubulovillous adenoma, stage I and stage IV CRC patients samples. Boxed regions indicate immune clusters with high resolution images below each whole tissue image. **(C–H)** Relative abundance of **(C)** 2539 m/z Hex7dHex1HexNAc6, **(D)** 2377 m/z Hex6dHex1HexNAc6, **(E)** 2852 m/z Hex7dHex1HexNAc6NeuAc1, **(F)** 2742 m/z Hex7dHex1HexNAc7, **(G)** 2012 m/z Hex5dHex1HexNAc5, and **(H)** 2,174 m/z Hex6dHex1HexNAc5 across polyp, tubulovillous adenoma, stage I and stage IV CRC tissues with corresponding representative MALDI-MSI image in the immune clusters. Intensity gradient from blue (least abundant) to red most abundant. Scale bars are below images. *p < 0.05, **p < 0.01, ***p < 0.001, ****p < 0.0001 using Kruskal-Wallis tests.

**FIGURE 5:**
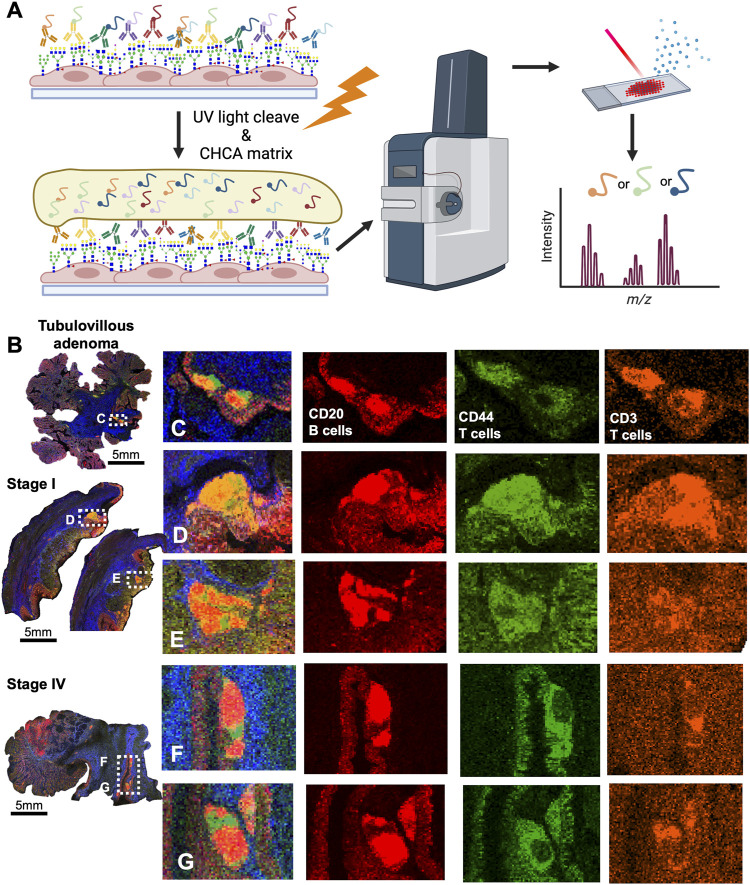
CRC immune clusters are comprised of CD20^+^ Bcells and CD44^+^, CD3^+^ T-cells as detected by MALDI-IHC. **(A)** Schematic of MALDI-IHC workflow demonstrating antibody saturation, matrix coating, and detection of the intensity of photo-cleaveable tags. **(B)** Whole tissue overview of MALDI-IHC for the tubulovillous adenoma, stage I, and stage IV CRC tissues. Boxed regions are the immune clusters with label indicating adjacent panel of high resolution images. Scale bars are below images. **(C)** High resolution image of the tubulovillous adenoma immune clusters with an overlay of the tags and individual images of CD20, CD44, and CD3 signatures by MALDI-IHC. **(D,E)** High resolution image of the stage I immune clusters with an overlay of the tag and individual images of CD20, CD44 and signatures by MALDI-IHC. **(F,G)** High resolution image of the stage IV immune clusters with an overlay of the tags and individual images of CD20, CD44 and CD3 signatures by MALDI-IHC. Colors indicated distinct photocleavable-tags.

Since CRC is associated with unique immune profiles leading to pathways of metastasis (Holst et al., 2015), we aimed to identify changes in the N-glycome of the immune clusters within the tumor microenvironment. Utilizing histopathology annotations, we observed lymphoid aggregates and follicles within a subset of our patient cohort. These clusters contain B cells, T cells and other immune supporting cells (Shah et al., 2013). Crucially, these clusters exhibit a distinctive N-glycan signature of 2,539 m/z Deeper investigation of these immune clusters by revealed a selection of N-glycans that may correlate with 2,539 m/z Hex7dHex1HexNAc6 (Figure 4A). Using histopathology guidance, we confirmed the significant increase of six N-glycans within these regions (Figure 4B). N-glycans of 2,539 m/z Hex7dHex1HexNAc6, 2,377 m/z Hex6dHex1HexNAc6, 2,852 m/z Hex7dHex1HexNAc6NeuAc1, 2,742 m/z Hex7dHex1HexNAc7, 2,012 m/z Hex5dHex1HexNAc5 and 2,174 m/z Hex5dHex1HexNAc5 were notably high in these regions with distinct spatial differences in terms of peak abundance (Figures 4C–H). Importantly, this signature is not influenced by the mucinous features of the tumor as indicated by co-registration of immune cells and pathological annotations. These findings suggest a unique feature associated with infiltrating immune cells that may have potential applications in therapeutics. Additional higher intensity signatures for 12 N-glycans but were notably variable in presence and intensity across tissues (Supplementary Figure S6). Finally, we sought to identify the cellular composition of these immune clusters by MALDI-IHC, a new multiplexed IHC method that uses photocleavable peptide tags detected by MALDI MSI (Figure 5A) (Yagnik et al., 2021). Performing traditional dewaxing, rehydration, antigen retrieval, overnight antibody incubation, and subsequent photocleavage of the light sensitive probes, we profiled the tubulovillous adenoma, stage I, and stage IV CRC tissues with immune specific antibodies of CD20 (B-cells), CD11b (monocytes), CD3 (T-cells), and CD44 (T-cells), and the extracellular matrix component of Collagen 1A1 (blue in whole tissue image) for structural information. Spatial differences were seen across tubulovillous adenoma, stage 1, and stage IV CRC tissues for all photocleavable tags with specific increased intensities in the immune clusters (Figure 5B). High resolution images of the immune clusters demonstrate heterogeneity of CD20, CD44, and CD3 photocleavable tags across the tissues (Figures 5C–G), with notable CD20 B-cells in the center surrounded by T-cells. This is indicative of germinal center formation (Trajkovski et al., 2018). Further investigation is needed to exactly identify the mechanism of evolution for these immune clusters in cellular composition through disease progression.

## Discussion

Previous research studies have investigated relative differences in N-glycan groups between normal and CRC tissues and cell lines. Increased expression of high-mannose and paucimannose groups has been observed in cancers relative to paired normal adjacent tissues (Balog et al., 2012; Sethi et al., 2015; Zhang et al., 2019; Coura et al., 2021) or normal tissue from healthy controls (Holm et al., 2020). Our results for paucimannose N-glycans are in line with these earlier findings, where we observed higher mean percentages of these glycans in cancers versus healthy normal controls. In addition, we observed a significant difference in paucimannose N-glycan expression between cancers and colorectal polyps. Kaprio and collegues also reported lower paucimannose glycan group expression in patients with preinvasive rectal lesions compared with invasive rectal CRCs. (Kaprio et al., 2015). Most studies have identified relatively higher high-mannose expression in CRCs versus paired normal where our results identified higher expression in normal and preinvasive lesions relative to CRCs. These differences could stem from using normal tissues from healthy donors (Holm et al., 2020). Our small sample size and mixed sample types might also contribute to these variations. However, similar to our results for adenomas, Boyaval et al. identified higher differential expression of high-mannose groups in preinvasive dysplastic regions compared to the invasive regions pointing to a possible decrease in high mannose glycosylation as lesions progress (Boyaval et al., 2022). This may reflect the overall differences in glucose metabolism in these tissues, whereby the adenomas are more metabolically active for glucose utilization, and therefore more substrate for protein glycosylation, than in the tumors. More data is needed to clarify the role of high-mannose in CRC tumor progression. In-depth analysis of the N-glycome of colorectal cancer cell lines revealed 139 N-glycans classified into paucimannosidic, oligo-mannosidic, complex, and hybrid groups, with additional segmentation into core/antennae fucosylation, sialylation, LacdiNAc motifs, Lewis type antigens, H blood group antigen, sulfation, and phosphorylation, accomplished by nano-liquid chromatography coupled to electrospray ionization mass spectrometry. This detailed analysis highlights the complexity of CRC cell lines, which can be extended to tissue samples (Sethi et al., 2015; Sethi and Fanayan, 2015).

We observed distinct stratification by N-glycome signatures, moving from normal to cancerous tissues, primarily driven by high mannose glycans. Furthermore, when we separated cancerous tissues into mucinous and nonmucinous samples, we identified distinct profiles in the intensity of high mannose, biantennary, and fucosylated N-glycans. Subsequently, our analysis of the tumor microenvironment revealed immunological features, and we identified a signature at 2,539 m/z Hex7HexNAc6Fuc1 associated with immune aggregates and follicles in proximity to the tumor. These findings demonstrate the importance of spatial information for N-glycome profiling of CRC tissues. Follow up studies will focus on differentiation of fucosylation isomers and 2,3/2,6 sialic acid linkages on the tri- and tetraantennary N-glycans (West et al., 2020; Lu et al., 2023). We will also perform focused analysis of immune cluster 2,3, and 2,6 sialylation using our recently reported chemical amidation workflow (Lu et al., 2023). In very preliminary analysis using this method, in 2 CRC tissues, the tumor and immune glycans were primarily 2,6 or mixed 2,6/2,3 isomers.

Multi-branched glycan groups, including tri- and tetraantennary structures are commonly associated with higher relative expression in serum of patients with CRC compared to controls, consistent with our results observed for tetraantennary branched glycans (de Vroome et al., 2018; Doherty et al., 2018; Coura et al., 2021). Coura and others observed higher expression in CRCs tissues for branched N-glycans compared to paired normal tissues (Coura et al., 2021). Several studies in colon and other cancers have found that biantennary N-glycans had higher expression in normal tissue relative to cancerous tissues (Zhao et al., 2018; Boyaval et al., 2022). However, we observed no differences in biantennary N-glycans between normal, polyp or cancerous tissues.

While we report a broad range of glycosylation changes that occur with progression to CRC, our study is limited by small sample sizes. Differences in patient-level and clinicopathologic characteristics of the samples may also play a role in the N-glycome of each group. Additionally, this data was collected in batches over a term of 3 years. This was accounted for by normalizing the N-glycan intensities to the total N-glycan signature of each sample and then represented as a relative value to the total to be compared between samples. This normalization method was reproducible across all samples. While we adjusted our analyses for patient age, there are indications that other demographic factors, such as sex, race, diet, obesity, or geography, may impact glycan signatures and abundances. Different molecular features in various types of colorectal polyps and CRCs are known to differ and could contribute to variations in N-glycan signatures across studies and sample types. Many studies, including ours, utilize a mix of sample types with varying pathologic features including size, histology, grade, stage, and CRC phenotypes. For example, the consensus molecular CRC phenotype-III (Guinney et al., 2015) has been found to overexpress carbohydrates (e.g., glucose, mannose) relative to the other CMS types pointing to the possibility that N-glycan patterns may differ by phenotype. CRC phenotypes also show wide variation in somatic, and epigenetic profiles, as well as immune signatures (Angelova et al., 2015). In fact, several new studies point to the importance of glycosylation in therapeutic immunotherapy resistance, which may offer opportunities for developing targeted anti-glycan drugs (Krug et al., 2023). Currently, only 15% of patients diagnosed with CRC are eligible for immunotherapy, greatly limiting improvements in survival for most advanced stage patients.

In summary, we provide a defined N-glycome spectrum across normal colon, adenoma, tumor associated poylps, and colorectal carcinoma tissue and immune clusters. It is possible to subdivide cancerous tissues by pathological designation of mucinous vs. nonmucinous tissues by the N-glycome of each tissue. Additionally, it is possible to detect N-glycan signatures within immune clusters when they arepresent in patient samples. These can then be correlated to cell compositions using multiplexed immune cell markers by MALDI-IHC. Our ability to detect specific N-glycan signatures in intra-polyp and intra-tumor immune cell populations combined with multiplexed MALDI-IHC could contribute to developing more effective CRC immunotherapies. Future studies will expand sample numbers, and ancestral origins to further define the immune cell N-glycans linked with outcomes.

## Data Availability

The raw data supporting the conclusion of this article will be made available by the authors, without undue reservation.

## Supplementary material

SUPPLEMENTAL FIGURE S1 N-glycans exhibit spatial differences across normal, polyp and CRC tissues. **(A)** H&E images of normal, polyp, tubulovillous adenoma (TVA), tumor associated polyp (TAP) annotated, nonmucinous, and mucinous CRC tissues. TVA was included to demonstrate malignant like features in this polyp. **(B)** Representative image of high mannose N-glycan 1419 m/z Hex6HexNAc2. **(C,D)** MALDI-MSI representative image of core fucose N-glycan **(C)** 1,444 m/z Hex4dHex1HexNAc3. **(D)** 2,100 m/z Hex5dHex1HexNAc4NeuAc1 and **(E)** 2,450 m/z Hex5dHex2HexNAc5NeuAc1. Glycan structures are placed on the left side of their corresponding images. Intensity gradient from blue (least abundant) to red most abundant. Scale bars are below the H&E images.

SUPPLEMENTAL FIGURE S2 Colorectal carcinoma tissue exhibits trending stage-dependent decreases in pauci-mannose N-glycans. **(A)** Relative Abundance of pauci-mannose N-glycans by stage. **(B)** H&E images of Stage I, II, and III/IV CRC patient tissues. **(C,D)** MALDI-MSI images of **(C)** 933 m/z Hex3HexNAc2, (D) 1,079 m/z Hex3dHex1HexNAc2, for Stage I, II, and III/IV CRC patient tissues. Glycan structures are placed on the left side of their corresponding images. Intensity gradient from blue (least abundant) to red most abundant. Scale bar is below the H&E images. **p* < 0.05, ***p* < 0.01, ****p* < 0.001, *****p* < 0.0001 using Kruskal-Wallis tests.

SUPPLEMENTAL FIGURE S3 Colorectal carcinoma tissue exhibits no stage-dependent changes in biantennary N-glycans. **(A)** Relative Abundance of biantennary N-glycans by stage. **(B)** H&E images of Stage I, II, and III/IV CRC patient tissues. **(C–F)** MALDI-MSI images of **(C)** 1,955 m/z Hex5dHex2HexNAc4, **(D)** 2,101 m/z Hex5dHex3HexNAc4, **(E)** 2,158 m/z Hex5dHex2HexNAc5, and **(F)** 2,449 m/z Hex5dHex2HexNAc5NeuAc1 for Stage I, II, and III/IV CRC patient tissues. Glycan structures are placed on the left side of their corresponding images. Intensity gradient from blue (least abundant) to red most abundant. Scale bar is below the H&E images. **p* < 0.05, ***p* < 0.01, ****p* < 0.001, *****p* < 0.0001 using Kruskal-Wallis tests.

SUPPLEMENTAL FIGURE S4 Colorectal carcinoma tissue exhibits trending stage-dependent increases in tetraantennary N-glycans. **(A)** Relative Abundance of tetraantennary N-glycans by stage. **(B)** H&E images of Stage I, II, and III/IV CRC patient tissues. **(C–F)** MALDI-MSI images of **(C)** 1891 m/z Hex3dHex1HexNAc6, **(D)** 2,987 m/z Hex7dHex4HexNAc6, **(E)** 3,051 m/z Hex8dHex2HexNAc7 and **(F)** 3,343 m/z Hex8dHex4HexNAc7 for Stage I, II, and III/IV CRC patient tissues. Glycan structures are placed on the left side of their corresponding images. Intensity gradient from blue (least abundant) to red most abundant. Scale bar is below the H&E images. **p* < 0.05, ***p* < 0.01, ****p* < 0.001, *****p* < 0.0001 using Kruskal-Wallis tests.

SUPPLEMENTAL FIGURE S5 Colorectal carcinoma tissue exhibits trending stage-dependent decreases in high mannose N-glycans. **(A)** Relative Abundance of high mannose N-glycans by stage. **(B)** H&E images of Stage I, II, and III/IV CRC patient tissues. **(C–F)** MALDI-MSI images of **(C)** 1,955 m/z Hex5dHex2HexNAc4, **(D)** 2,101 m/z Hex5dHex3HexNAc4, **(E)** 2,158 m/z Hex5dHex2HexNAc5 and **(F)** 2,449 m/z Hex5dHex2HexNAc5NeuAc1 for Stage I, II, and III/IV CRC patient tissues. Glycan structures are placed on the left side of their corresponding images. Intensity gradient from blue (least abundant) to red most abundant. Scale bar is below the H&E images. **p* < 0.05, ***p* < 0.01, ****p* < 0.001, *****p* < 0.0001 using Kruskal-Wallis tests.

SUPPLEMENTAL FIGURE 6 Unique N-glycan signatures to tumor associated immune aggerates in CRC patient samples. **(A–L)** MALDI-IMS images of **(A)** 1257 m/z Hex5HexNAc2, **(B)** 1,419 m/z Hex6HexNAc2 , **(C)** 1581 m/z Hex7HexNAc2, **(D)** 1743 m/z Hex8HexNAc2, **(E)** 1,905 m/z Hex9HexNAc2, **(F)** 1,866 m/z Hex5HexNAc5, **(G)** 2,067 m/z Hex10HexNAc2, **(H)** 2,231 m/z Hex6HexNAc6, **(I)** 2377m/z Hex6dHex1HexNAc6, **(J)** 2,487 m/z Hex6dHex1HexNAc5NeuAc1 + 2Na, **(K)** 2,905 m/z Hex8dHex1HexNAc7, and **(L)** 3,270 m/z Hex9dHex1HexNAc8 for CRC patient tissues. Glycan structures are placed on the left side of their corresponding images. Intensity gradient from blue (least abundant) to red most abundant. Scale bar is below images.
