# Supplementary figures and images for: Utilizing multimodal mass spectrometry imaging for profiling immune cell composition and N-glycosylation across colorectal carcinoma disease progression
Source: Front Pharmacol. 2024 Jan 11;14:1337319. doi: 10.3389/fphar.2023.1337319 (PMC10808565; doi:10.3389/fphar.2023.1337319)

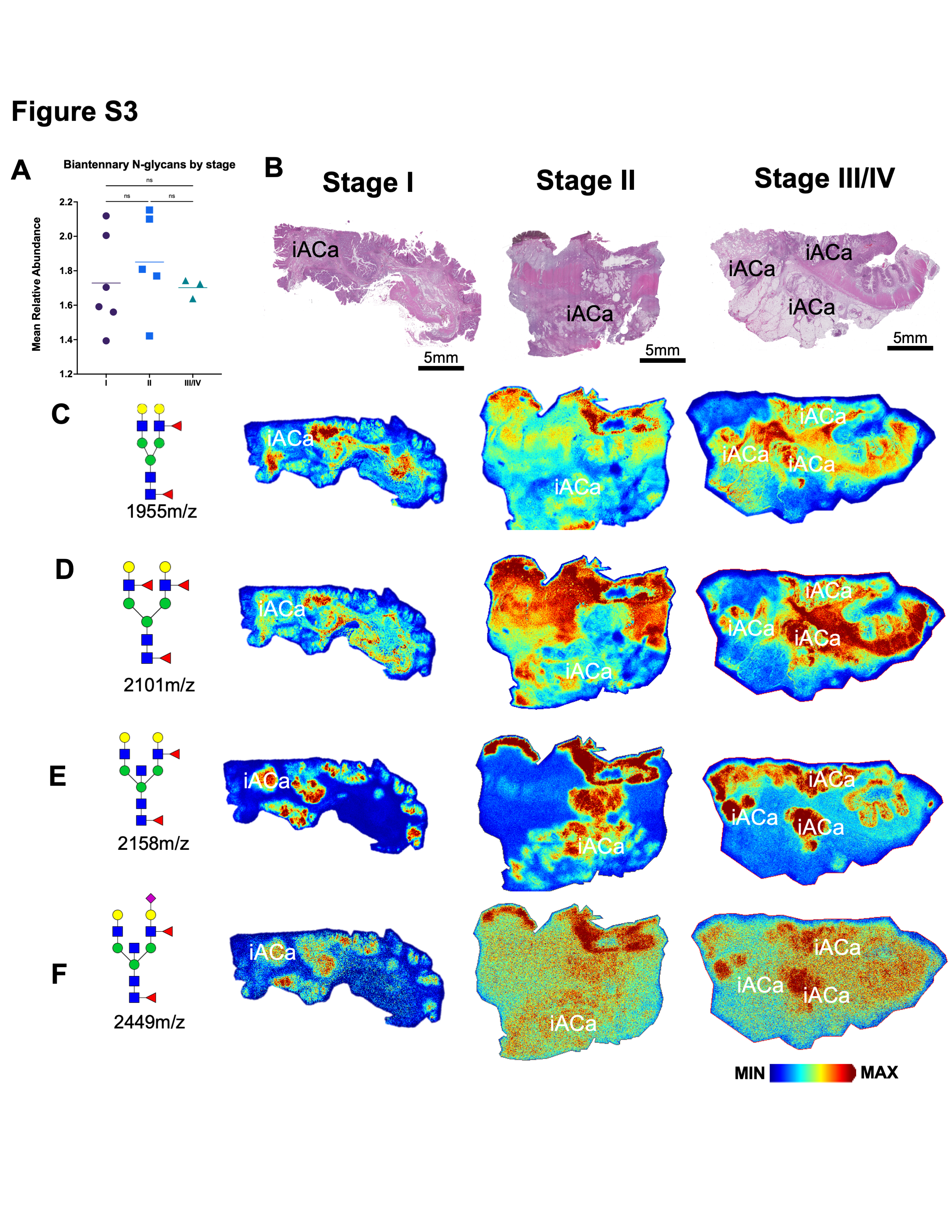

Supplement: Supplementary file 1 [file Image3.TIFF]

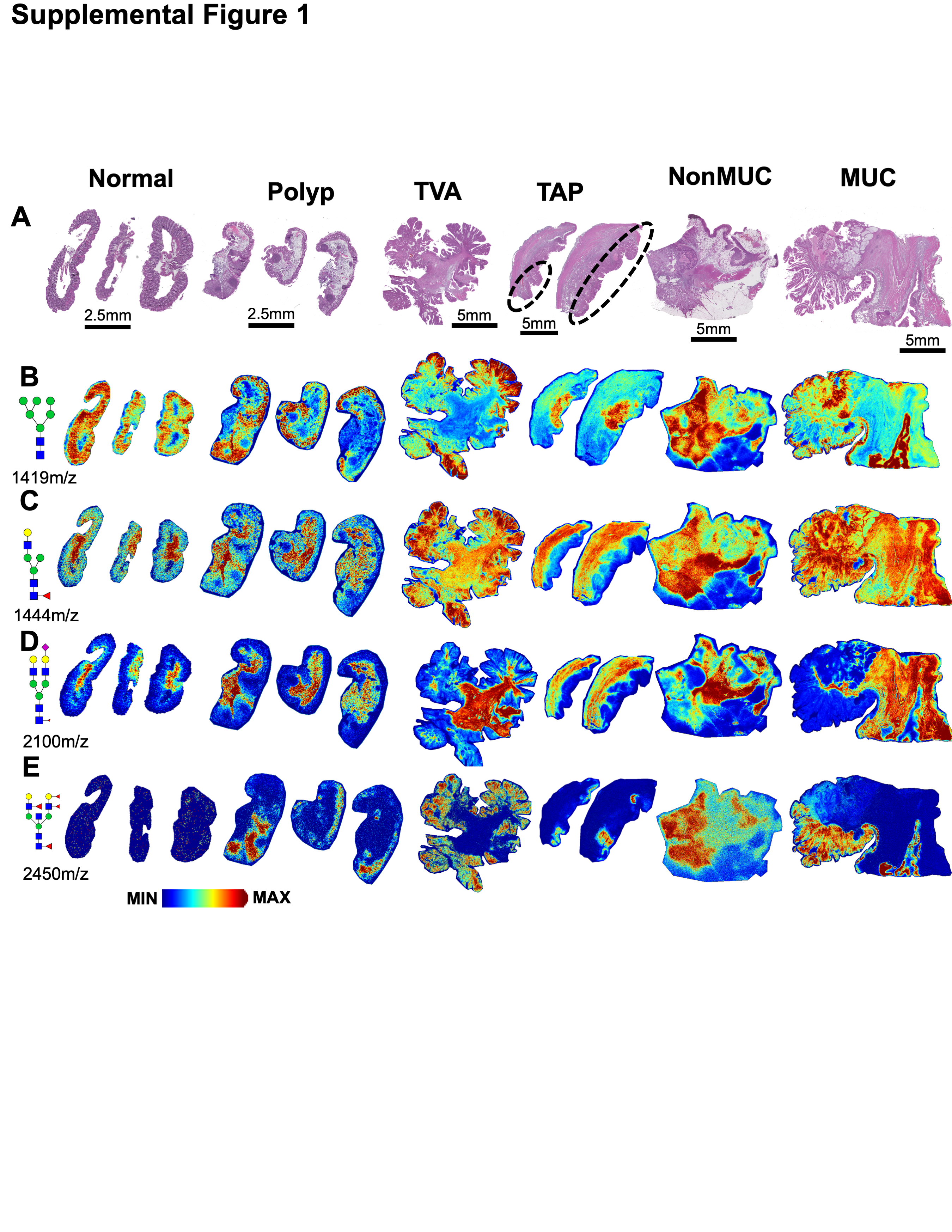

Supplement: Supplementary file 2 [file Image1.TIFF]

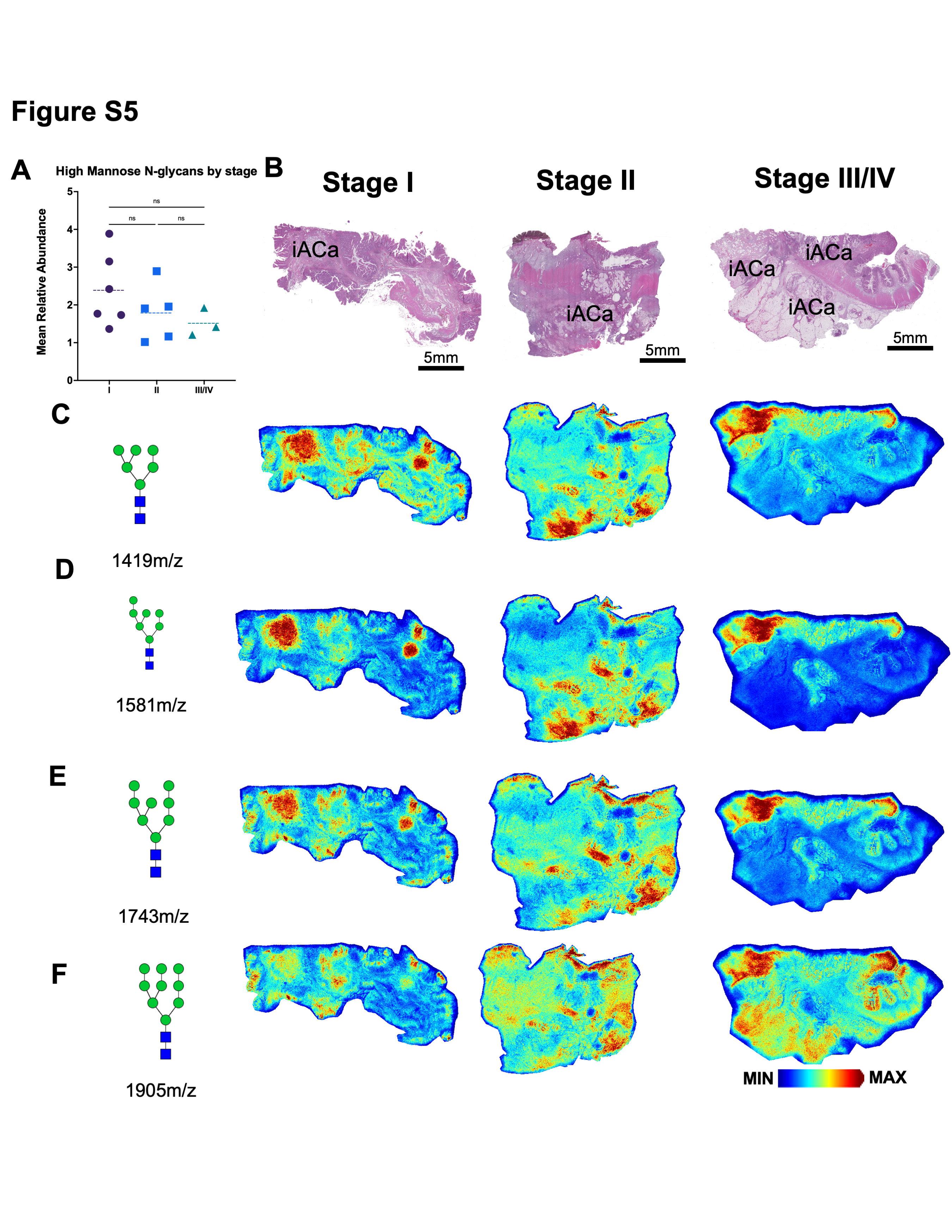

Supplement: Supplementary file 3 [file Image5.TIFF]

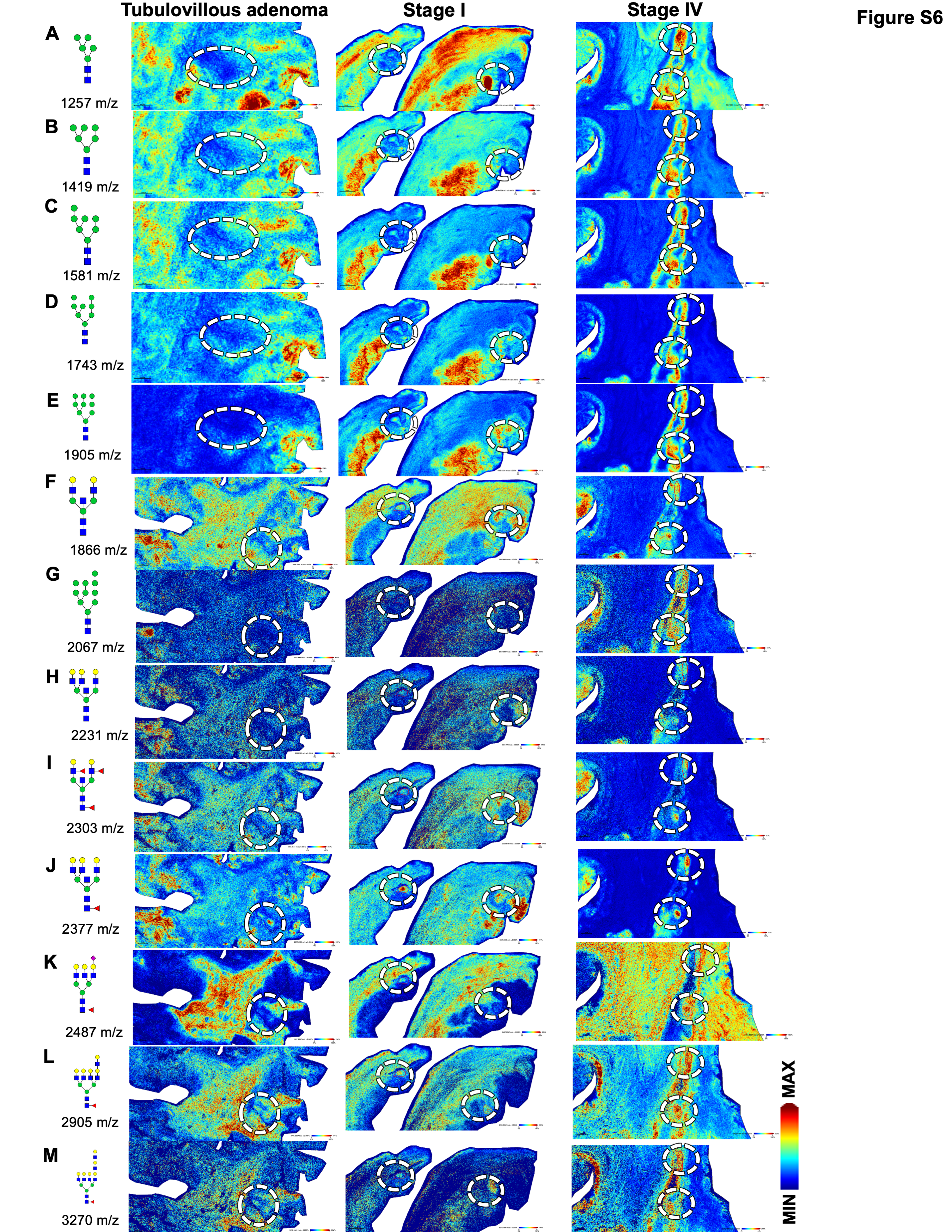

Supplement: Supplementary file 5 [file Image6.TIFF]

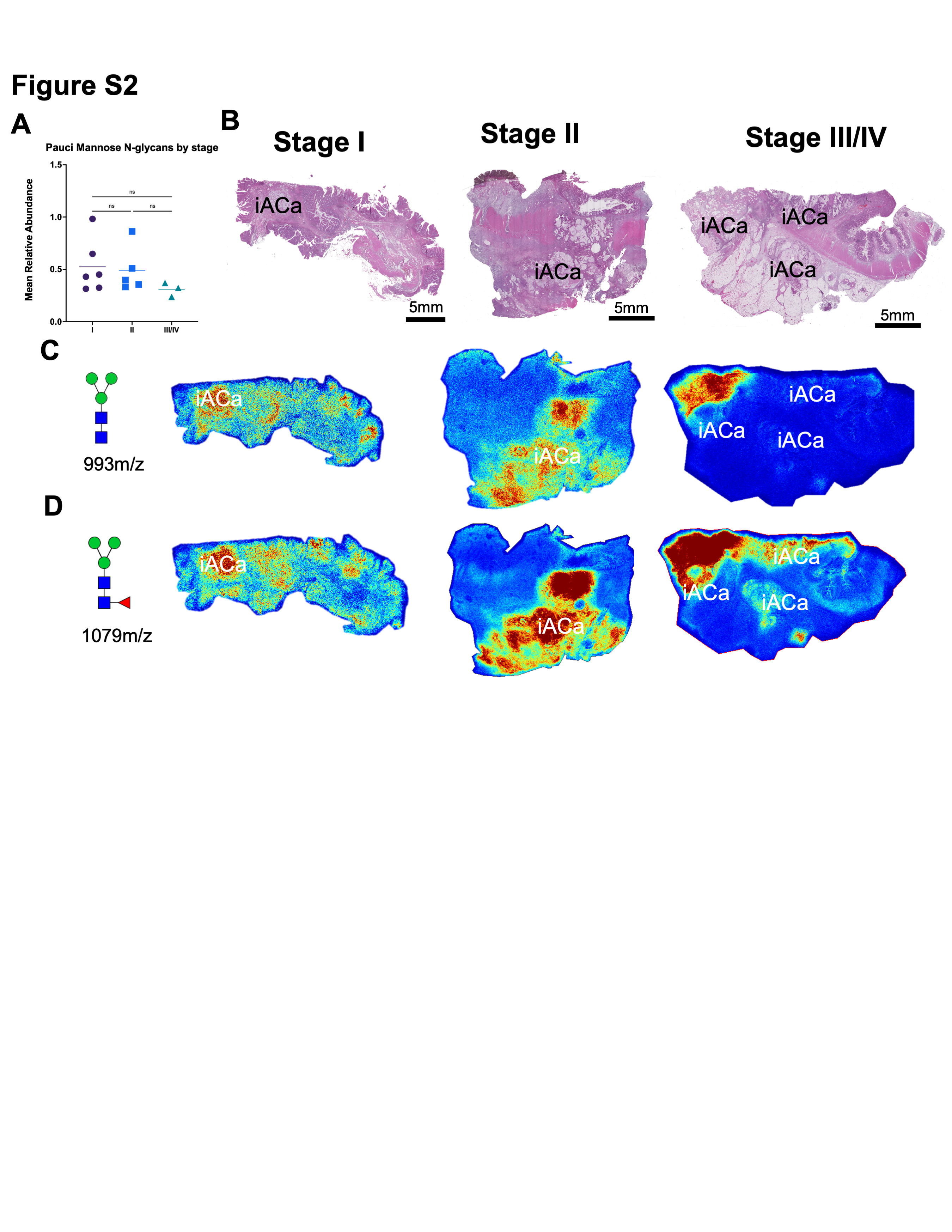

Supplement: Supplementary file 6 [file Image2.TIFF]

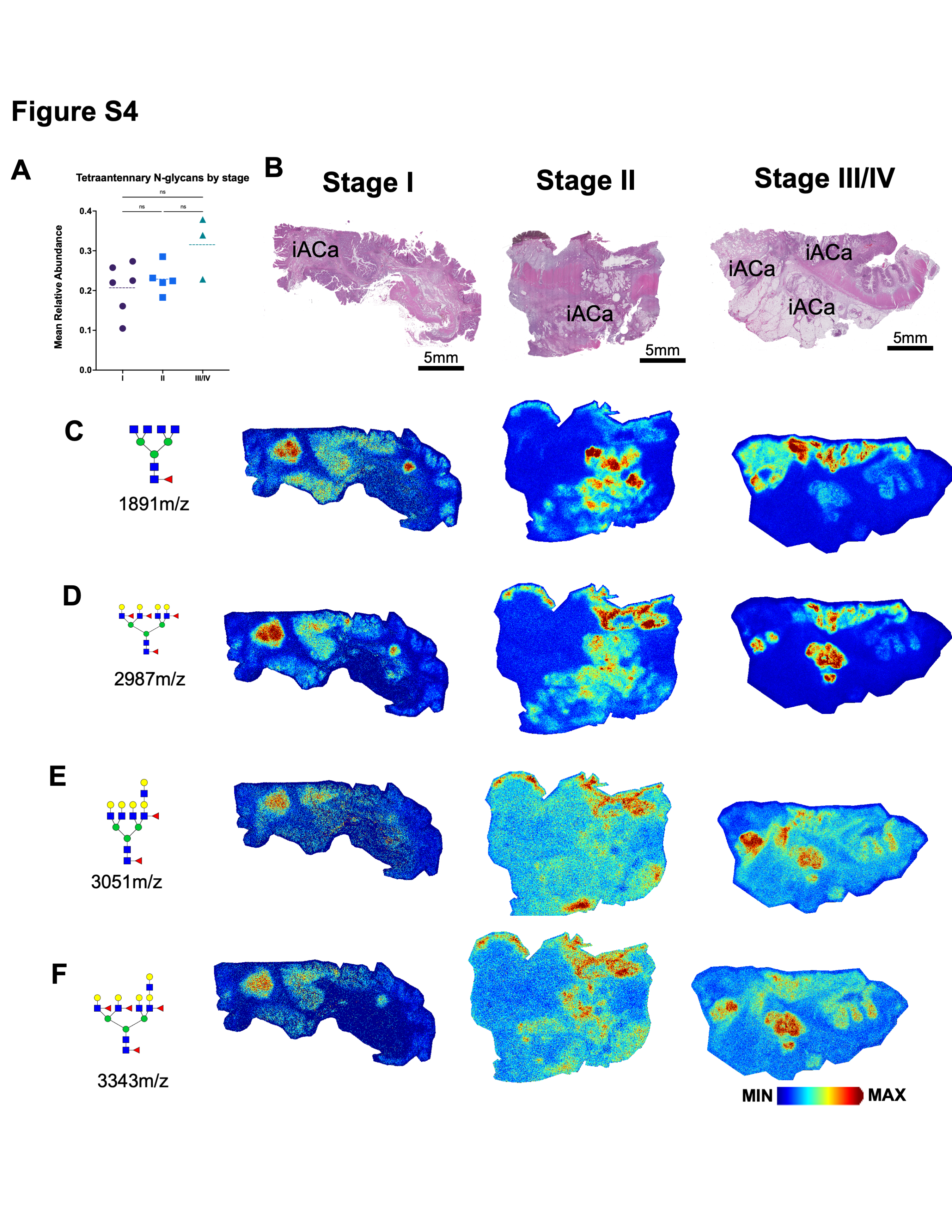

Supplement: Supplementary file 7 [file Image4.TIFF]
